# SOCS6 Promotes Mitochondrial Fission and Cardiomyocyte Apoptosis and Is Negatively Regulated by Quaking-Mediated miR-19b

**DOI:** 10.1155/2022/1121323

**Published:** 2022-01-27

**Authors:** Peng Zhang, Ping Guan, Xiaomiao Ye, Yi Lu, Yanwen Hang, Yanling Su, Wei Hu

**Affiliations:** Department of Cardiology, Minhang Hospital, Fudan University, Shanghai, China

## Abstract

**Background:**

Mitochondrial dysfunction and abnormal mitochondrial fission have been implicated in the complications associated with I/R injury as cardiomyocytes are abundant in mitochondria. SOCS6 is known to participate in mitochondrial fragmentation, but its exact involvement and the pathways associated are uncertain.

**Methods and Results:**

The expression of SOCS6 was analyzed by western blot in cardiomyocytes under a hypoxia and reoxygenation (H/R) model. A dual-luciferase reporter assay was used to confirm the direct interaction between miR-19b and the 3′-UTR of Socs6. In the present study, we found that Socs6 inhibition by RNA interference attenuated H/R-induced mitochondrial fission and apoptosis in cardiomyocytes. A luciferase assay indicated that Socs6 is a direct target of miR-19b. The overexpression of miR-19b decreased mitochondrial fission and apoptosis *in vitro*. Moreover, the presence of miR-19b reduced the level of SOCS6 and the injury caused by I/R *in vivo*. There were less apoptotic cells in the myocardium of mice injected with miR-19b. In addition, we found that the RNA-binding protein, Quaking (QK), participates in the regulation of miR-19b expression.

**Conclusions:**

Our results indicate that the inhibition of mitochondrial fission through downregulating Socs6 via the QK/miR-19b/Socs6 pathway attenuated the damage sustained by I/R. The QK/miR-19b/Socs6 axis plays a vital role in regulation of mitochondrial fission and cardiomyocyte apoptosis and could form the basis of future research in the development of therapies for the management of cardiac diseases.

## 1. Introduction

Through advances in disease management, the mortality rate associated with myocardial infarction (MI) has declined worldwide [1, 2]. However, the reperfusion strategies used to treat MI can result in ischemia/reperfusion (I/R) injury, which can cause further complications such as chronic heart failure and myocardial dysfunction [3–6]. Several studies have implicated mitochondrial dysfunction and abnormal mitochondrial fission in the complications associated with MI and I/R injury [7–9]. High-energy-requiring cardiomyocytes are abundant in mitochondria, which occupy 30% of the total cell volume, and are therefore vulnerable to cellular changes that would impact mitochondrial function [10]. The inhibition of functional changes in the mitochondria may prevent some of the complications associated with I/R injury.

Changes in the morphology of mitochondria occur frequently, mainly through either fusion or fission and in response to the cellular environment [11]. Fusion enables adjacent mitochondria to combine and elongate whereas fission enables an equal division of mitochondria during cell division but also the fragmentation and the removal of damaged mitochondria by mitophagy [12]. Disrupting the balance between fusion and fission by factors such as oxidative stress and ischemia is believed to increase mitochondrial permeability and promote apoptosis, thereby making the heart more vulnerable to I/R injury [13]. Transplantation of autologously derived mitochondria into the heart of rabbits before the induction of I/R was found to enhance the consumption of oxygen and cardiac function postinjury [14]. Mitochondrial fission is controlled by dynamin-related protein 1 (DRP1); P110, a selective peptide inhibitor of DRP1, administered to rats before I/R was able to prevent excessive mitochondrial fragmentation and improve mitochondrial oxygen consumption [15]. Mutations in DRP1 prevent fission and lead to an excessive number of fused mitochondria [16]. Ser637 in DRP1 is a cyclic AMP-dependent kinase phosphorylation site that prevents DRP1 from being translocated from the cytosol to mitochondria; however, calcineurin-dependent dephosphorylation of this site increases translocation [17]. Suppressor of cytokine signaling 6 (SOCS6) is known to inhibit the phosphorylation of DRP1 thereby promoting its mitochondrial translocation [18]. Consequently, upregulated SOCS6 induces mitochondrial fragmentation whereas its downregulation increases mitochondrial fusion.

Recently, there has been interest in the epigenetic mechanisms which control the expression of genes in coronary heart disease and mitochondria [19, 20]. Quaking (QKI in humans, QK in mice) is a member of the signal transduction and activation of RNA (STAR) family of RNA-binding proteins (RBPs) involved in various forms of epigenetic processing, such as pre-mRNA splicing and controlling the stability and turnover of microRNA (miRNA) [21, 22]. RBPs such as QKI recognize *cis* elements in 3′ untranslated regions (UTR) and are responsible for the posttranscriptional regulation of multiple functions including maintaining the endothelial barrier, regulating smooth muscle, and responding to inflammatory stimuli [23, 24]. Bioinformatic analysis predicted that the 3′-UTR of Socs6 mRNA contained a putative target site for miR-19b, which is highly conserved in mammals and among the miRNA specifically regulated by QKI [22, 25]. The miR-19b expression levels are decreased when QKI is silenced. Moreover, there is known to be a relationship between miR-19b and patients who have ST-elevated MI and miR-19b is downregulated in the infarct area of a murine model of I/R injury [26, 27].

In the present study, we assess the involvement of mitochondrial dynamics in I/R injury by using a hypoxia and reoxygenation (H/R) model in cardiomyocytes and a murine model of I/R. In particular, we examine the roles of Socs6, QK, and miR-19b expression on levels of mitochondrial fission and apoptosis *in vitro* and *in vivo*. Finally, we review the literature associated with the QK/miR-19b/Socs6 axis and discuss whether it may have potential in the improved management of cardiac diseases.

## 2. Materials and Methods

### 2.1. Cell Cultures and H/R Treatment

Cardiomyocytes from a mouse HL-1 cardiac cell line (American Type Culture Collection (ATCC), Manassas, VA, USA) were cultured in Claycomb media with 10% fetal bovine serum (FBS, Gibco), 0.1 mmol/L noradrenaline, 2 mmol/L L-glutamine, and penicillin/streptomycin (Invitrogen, Carlsbad, CA, USA) at 37°C in 5% CO_2_. To induce H/R, cells were exposed to 1% O_2_, 94% N_2_, and 5% CO_2_ for 2 h (hypoxia) followed by 95% air and 5% CO_2_ for 12 h (reoxygenation). Human embryonic kidney cell (HEK293T, ATCC) was cultured in Dulbecco's modified Eagle's medium with 10% FBS.

### 2.2. Western Blot Analysis

Cells and heart tissues for western blotting were first lysed in RIPA buffer (Beyotime, Jiangsu, China). Mitochondrial fractions were isolated using the Mitochondria Isolation Kit, based on the manufacturer's instructions (PIERCE, Rockford, IL, USA). Proteins in the lysate (30 *μ*g) were separated using SDS-PAGE and then transferred to PVDF membranes. The membranes were blocked in 5% skimmed milk and 0.1% Tween-20 for 1 h and then incubated with primary antibodies at 4°C overnight. The primary antibodies and dilutions used in this study were anti-SOCS6 (1 : 1000, Abcam, Cambridge, UK), anti-DRP1 (1 : 1000, Abcam), anti-DRP1 (phospho S637) (1 : 500, Abcam), anti-QK (1 : 1000, Cell Signaling Technology, Boston, MA, USA), anti-VDAC1 (1 : 1000, Abcam), and anti-*β*-actin (1 : 5000; Sigma-Aldrich, St. Louis, MO, USA). Membranes were either probed with horseradish peroxidase-conjugated with goat anti-rabbit IgG or goat anti-mouse IgG (Santa Cruz Biotechnology, Dallas, TX, USA). Final blots were visualized by an Amersham ECL Western Blotting Detection Reagent (GE Healthcare, Amersham, UK). The intensity of protein bands was quantified by ImageJ software.

### 2.3. Quantitative Reverse Transcription PCR

Trizol was used to extract total RNA extracted from cells (Thermo Fisher Scientific, Waltham, MA, USA). Revere transcription (RT) was performed on 2 *μ*g of RNA to produce cDNA using HiScript Reverse Transcriptase (RNase H; Vazyme, Nanjing, China). Quantitative PCR was performed using SYBR Green Master Mix (Vazyme) on a QuantStudio 6 Flex Real-Time PCR System (Applied Biosystems, Foster City, CA, USA). The level of miRNA and mRNA expression was normalized to U6 and GAPDH expression, respectively. The relative expression of genes was presented as fold change and was calculated using the 2^−*ΔΔ*CT^ method.

### 2.4. Lactate Dehydrogenase (LDH) Assay, MTT Assay, TUNEL, and Analysis of Mitochondrial Fission

LDH concentrations were measured in the supernatant of cell cultures after H/R to assess leakage. Cell culture supernatants were harvested, and LDH content was measured using an LDH kit (Roche, Mannheim, Germany) according to the manufacturer's instructions.

Total cell death was assessed using MTT cell viability assay. Cells were initially seeded in a 96-well plate. After 24 h of plating, the medium was replaced with 0.5 mg/mL MTT in serum-free and phenol-red free medium for 4 h. Then, the medium containing MTT was removed and 75 *μ*L DMSO solution was added to stop the reaction and dissolve the crystals for 10 min at 37°C. The resulting formazan blue dye was measured using a spectrophotometer at 570 nm, which thus provides a value for the proportion of number of live cells.

The level of apoptosis was determined in cells following H/R by using terminal deoxynucleotidyl transferase-mediated dUTP nick end labeling (TUNEL). Following H/R, cells were cultured on coverslips and fixed in 4% paraformaldehyde for 5 min, and then, the TUNEL procedure was performed using a TUNEL kit (Roche, Hamburg, Germany). Nuclei were counterstained with DAPI. The percentage of TUNEL-positive cells was determined from a mean of at least 10 fields under a confocal laser scanning microscope (Nikon C2, Nikon Corporation, Tokyo, Japan).

To determine mitochondrial morphology, cardiomyocytes plated onto collagen-treated coverslips were stained with MitoTracker Red CMXRos (Molecular Probes, Eugene, OR, USA) for 20 min. The percentage of cells with fragmented mitochondria from at least 300 cells per group was determined under a confocal laser scanning microscope (Nikon C2) and presented as the mean ± SEM.

### 2.5. Luciferase Reporter Assay

To validate the interaction between Socs6 and miR-19b, we first transfected HEK293T cells with either a wild-type or mutant construct of mouse Socs6 with miR-19b mimic or anti-miR-19b. The 3′-UTR of Socs6 was amplified by PCR using the forward primer 5′-GGGGTACCCATGTTGGGGTAAGGAAGTCTCA-3′ and the reverse primer 5′-CCGCTCGAGGCGCGACATACTGTATCTAGAAG-3′ and subcloned into KpnI- and XhoI-digested pGL3-basic vector. Mutations were created using a QuikChange II XL Site-Directed Mutagenesis Kit (Stratagene, La Jolla, CA, USA). Dual-Luciferase Reporter Assay System (Promega, Madison, WI, USA) was used to measure the luciferase activity after 48 h (following manufacturer's instructions).

### 2.6. Lentiviral Constructions, Plasmid, and Oligonucleotide Transfection

Lentiviral constructs were designed to knockdown Socs6 and QK. Socs6 short hairpin RNAs (ShRNA) (5′-GCAGAATAACCCAATCCAAAG-3′) and QK ShRNA target sequence (5′-GCTGATGGAGCTTGCAATTCT-3′) were cloned into GV248 vectors (Genechem, Shanghai, China). A nonsilencing scrambled ShRNA was used as a negative control. Using HEK293T cells, lentiviral particles were produced. Further, lentiviral stocks were made after concentrating the samples using ultracentrifugation.

For Socs6 overexpression, mouse Socs6 CDS was amplified with the primers 5′-CTGGGATCCGCGCGATGAAGAAAATCAGTCTG-3′ (forward) and 5′-CAGCTCGAGTCAGTAGTGCTTCTCCTGCAAA-3′ (reverse), subcloned into pcDNA3.1 using BamH1 and XhoI restriction sites and transfected into cells using Lipofectamine 2000. pcDNA3.1 empty vector (EV) was used as a control. For miR-19b expression experiments, miR-19b and anti-miR-19b oligonucleotides and their corresponding controls (miR-NC and anti-miR-NC) were obtained from GenePharma (Shanghai, China). Lipofectamine 2000 was used for their successive transfection into the cells.

### 2.7. *In Vivo* Model of I/R

The use of animals in all experiments followed the established guidelines published by the National Institutes of Health (1996) following scientific, humane, and ethical principles on the use and care of laboratory animals for biomedical research. Animal experiments were approved by the Ethical Committee of Minhang Hospital, Fudan University. To induce myocardial I/R injury, C57BL/6 male mice from the Shanghai SLAC Laboratory Animal Center (Shanghai, China) were anesthetized by 5% isoflurane. Anesthetization was maintained at 1.5–2% in a 100% oxygen flow, and body temperature of 37°C was maintained throughout the surgery. The heart was first exposed, and then, using the 8-0 silk ligature, the left anterior descending coronary artery was ligated for 30 min occlusion. The ligature was then released and reperfusion occurred for 3 h; the analysis for cell apoptosis assay and mitochondrial morphology/function assessment were performed at this time point. Alternatively, at 24 h, infarct size determination was performed. Following reperfusion for the times indicated, the mice were euthanized (CO_2_ inhalation) and hearts were recovered for further analysis.

### 2.8. *In Vivo* Delivery of Lentivirus and miRNA

Mice were anesthetized as described in Section 2.7. A microcatheter was introduced through an incision made in the middle of the neck, into the right common carotid artery. Lentivirus expressing Sh-Socs6 (1 × 10^7^ PFU) was injected (100 *μ*L) via the microcatheter. The microcatheter was then removed and the incision closed. To inject miR-19b mimic into the hearts, the hearts were exposed in mice anesthetized as described in Section 2.7. A microsyringe with a 30 G needle was inserted 1 mm deep into the left ventricular wall, and 5 *μ*g of miR-19b mimic was delivered. The chest was closed, and the mice were allowed to recover. Myocardial I/R injury was induced 72 hours after the injection of lentivirus or miR-19b mimic into the hearts.

### 2.9. Measurement of Myocardial Infarct Size and Apoptosis

To measure the impact of I/R on infarct size, hearts were first divided into five biventricular sections of equal thickness and then stained with 1.5% triphenyltetrazolium chloride (TTC, Sigma-Aldrich) solution for 15 min at 37°C. The unstained regions represented infarcted tissue and were quantified under a digital camera with NIH image software. To determine myocardial apoptosis, paraffin-embedded tissue was cut into 4–5 *μ*m sections and incubated in 50 *μ*L of TUNEL solution. The percentage of TUNEL-positive nuclei was calculated and defined as the apoptotic index.

### 2.10. Transmission Electron Microscopy

Tissue sections (1–2 mm) were cut perpendicular to the long axis of the left ventricular wall of the hearts isolated from mice. They were incubated overnight in 4% glutaraldehyde before being fixed in 1% osmium tetroxide for 1 h. Dehydration of the sections was carried out using a graded ethanol immersion series and embedded in resin. An ultramicrotome was then used to cut the resin-embedded tissue into 80 nm thick sections. The sections were placed under a JEM-1400 electron microscope (JEOL, Tokyo, Japan), and the images were captured with a CCD camera (Olympus, Tokyo, Japan). The TEM allowed mitochondria to be observed at ×10,000 magnification.

### 2.11. Mitochondrial Staining

The mitochondrial fission was assessed by staining the mitochondria using 20 *μ*M MitoTracker Red (Molecular Probes). Using confocal microscopy, we imaged the morphology of mitochondria and analyzed them individually. If 90% of the tubular mitochondria was disintegrated, then the mitochondrion was scored as fragmented. Such assessment was performed for at least 200 individual random cells before calculating the percentage of fragmented cells among the total 200 counted cells.

### 2.12. Statistical Analysis

Student's *t-*test was used to compare samples between two groups, and to compare samples among multiple groups, one-way analysis of variance (ANOVA) with Bonferroni's post hoc test was used. All the data are presented as the mean ± SEM of three independent experiments and a value of *P* < 0.05 was considered significant.

## 3. Results

### 3.1. SOCS6 Regulates H/R-Induced Mitochondrial Fission and Cardiomyocyte Apoptosis *In Vitro*

**Figure 1:**
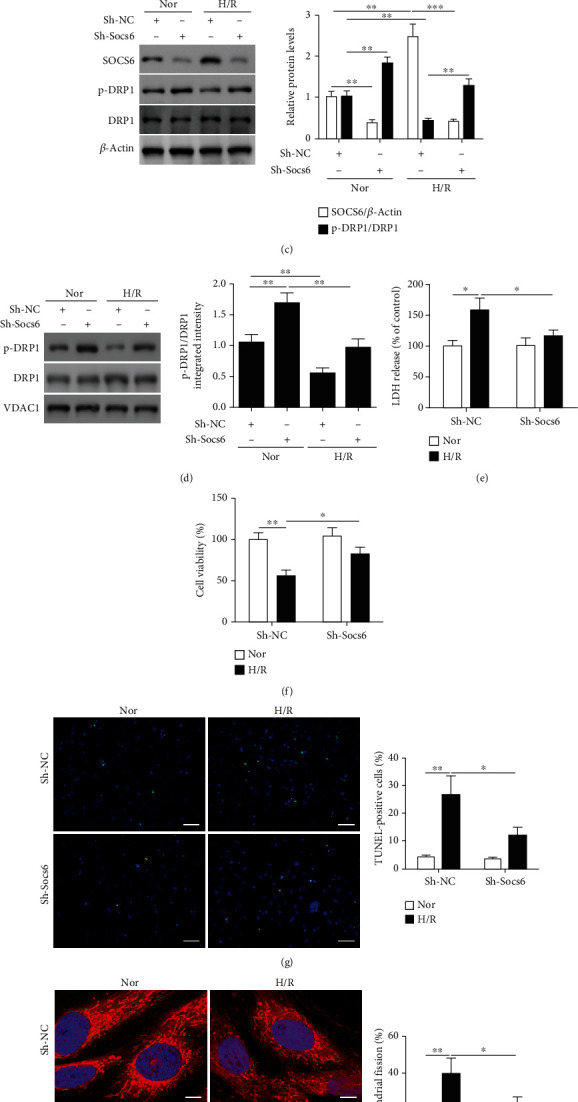
SOCS6 regulates mitochondrial fission and apoptosis in HL-1 cardiomyocytes. (a) HL-1 cardiomyocytes were treated with hypoxia and reoxygenation (H/R) at the times indicated. SOCS6 and phosphorylated DRP1 levels were analyzed by western blot assays. (b) HL-1 cardiomyocytes were infected with lentiviral constructs of Socs6 ShRNA or a negative control (Sh-NC). Socs6 mRNA levels were analyzed by qRT-PCR. HL-1 cardiomyocytes were infected with lentiviral constructs of Socs6 ShRNA or a negative control (Sh-NC) and then exposed to H/R. SOCS6 and phosphorylated DRP1 levels in (c) whole cell lysates and in (d) mitochondrial fractions were analyzed by western blot assays. (e) The release of lactate dehydrogenase (LDH) in HL-1 cardiomyocytes was assayed. (f) Total cell death was assessed using MTT cell viability assay. (g) Representative images of apoptosis analyzed by TUNEL staining, TUNEL-positive cells were counted and calculated (TUNEL: green; DAPI: blue). Scale bar, 50 *μ*m. (h) Representative confocal images of mitochondrial morphology stained with MitoTracker Red and the percentage of cardiomyocytes with fragmented mitochondria are shown. Nuclei were visualized by DAPI (MitoTracker Red: red; DAPI: blue). Scale bar, 10 *μ*m. ^∗^*P* < 0.05, ^∗∗^*P* < 0.01, and ^∗∗∗^*P* < 0.001.

To determine the effects of SOCS6 on levels of mitochondrial fission, we first examined mitochondrial morphology with different expression levels of SOCS6 in cardiomyocytes under H/R. Western blot analysis revealed that SOCS6 levels increased in cardiomyocytes following H/R compared to normoxic conditions, whereas the levels of DRP1 phosphorylation decreased (Figure 1(a)). However, when Socs6 is downregulated by ShRNA (Figure 1(b)), levels of DRP1 phosphorylation increased in whole cell lysates (Figure 1(c)) and mitochondrial fractions (Figure 1(d)). Additionally, it was also evident that the pDrp1/Drp1 levels were significantly higher when Socs6 was downregulated. Cardiomyocytes were assessed for injury using an assay to detect LDH leakage from cells. The release of LDH was significantly higher in cells exposed to H/R compared with control; however, cells transfected with Socs6 ShRNA and subjected to H/R released significantly less LDH (*P* < 0.05), indicating less damage (Figure 1(e)). Using MTT assay, we assessed the cell viability of cardiomyocytes under H/R in the presence or absence of Socs6 ShRNA. Evidentially, H/R significantly decreased cell viability but downregulation of Socs6 could significantly rescue this defect and increase the number of viable cells (Figure 1(f)). An assessment of apoptotic cells using TUNEL confirmed that H/R was severely detrimental to the number of viable cardiomyocytes (*P* < 0.01). However, downregulating Socs6 resulted in significantly fewer TUNEL-positive cells (*P* < 0.05) (Figure 1(g)). An analysis of mitochondrial morphology using confocal images and MitoTracker Red indicated a reduced percentage of fragmented mitochondria in cardiomyocytes following H/R with Socs6 silenced (Figure 1(h)). These results demonstrate that SOCS6 may be involved in the regulation of mitochondrial fission and apoptosis in HL-1 cardiomyocytes.

### 3.2. SOCS6 Inhibition Attenuates Myocardial Infarction and Apoptosis *In Vivo*

**Figure 2:**
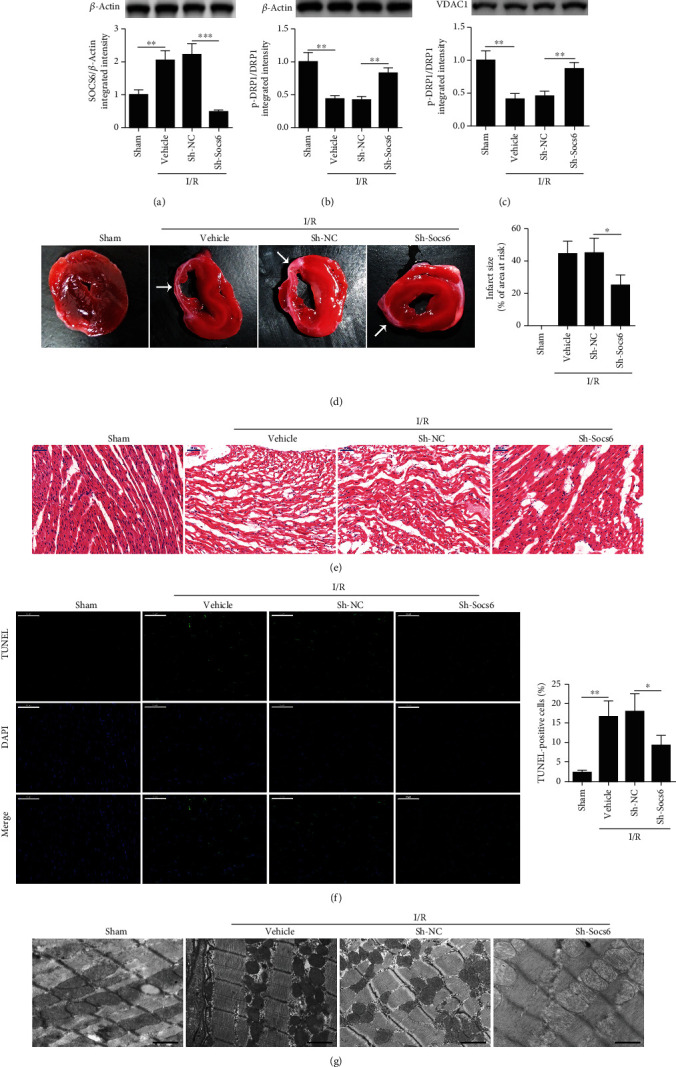
Socs6 inhibition attenuates myocardial infarction and apoptosis in mice. (a) Hearts of adult male C57BL/6 mice were injected with lentivirus expressing Sh-Socs6 or a negative control (Sh-NC). After 72 h, they were exposed to 30 min of myocardial ischemia (MI) followed by indicated time of reperfusion (*n* = 6 mice per group). SOCS6 levels were detected by western blotting. Phosphorylated DRP1 levels in (b) whole heart and in (c) mitochondrial fractions were analyzed by western blot assays. VDAC1 as a mitochondrial marker. (d) Representative sections of MI assayed by TTC staining after 24 h of reperfusion. The white arrow indicated the infarcted area. The infarct size is presented as the percentage area at risk. (e) Representative images of heart sections stained with hematoxylin and eosin. Scale bar, 50 *μ*m. (f) Apoptotic cells were detected by TUNEL assay after 3 h of reperfusion. Scale bar, 50 *μ*m. (g) Images of mitochondrial morphology obtained by transmission electron microscopy after 3 h of reperfusion. Scale bar, 1 *μ*m.

We next investigated whether the inhibition of Socs6 could attenuate MI and apoptosis *in vivo*. The hearts of adult male C57BL/6 mice were injected with lentivirus containing Sh-Socs6 or a negative control and then exposed to MI followed by reperfusion. Western blot analysis confirmed that levels of SOCS6 were reduced in mice with downregulated Socs6 following I/R (Figure 2(a)), and inhibition of Socs6 attenuated I/R-induced suppression of the levels of DRP1 phosphorylation in heart samples (Figure 2(b)) and in mitochondrial fractions (Figure 2(c)). The ratio of pDRP1/DRP1 was significantly higher among both samples after inhibition of Socs6. The infarct sizes in sections of myocardia following MI were significantly smaller in hearts with downregulated Socs6 (Figures 2(d) and 2(e)). The myocardium of mice with Socs6 silenced appeared healthier with less apoptotic cells, as confirmed in a TUNEL assay (Figure 2(f)). Moreover, TEM images of mitochondrial morphology in heart tissue sections with Socs6 silenced revealed lower fragmentation (Figure 2(g)), confirming that the inhibition of Socs6 can attenuate MI and apoptosis in an animal model of I/R injury.

### 3.3. miR-19b Represses Socs6 Expression and Participates in the Regulation of Mitochondrial Fission and Apoptosis

**Figure 3:**
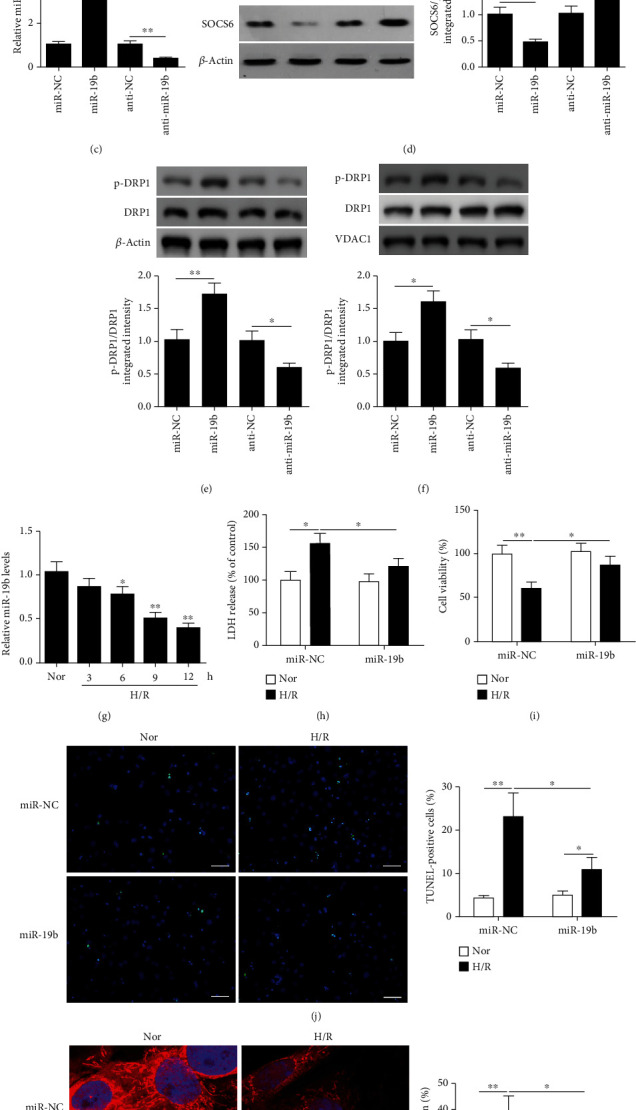
miR-19b represses Socs6 expression and participates in the regulation of mitochondrial fission and apoptosis. (a) The putative target site of mmu-miR-19b-3p located in the 3′-UTR of Socs6 mRNA as predicted by TargetScan software. (b) Luciferase activity was determined 48 h after HEK293T cells were cotransfected with wild-type or mutated 3′-UTR of mouse Socs6 with miR-19b mimic or anti-miR-19b. (c) Relative miR-19b levels in HL-1 cells transfected with miR-19b mimic, anti-miR-19b, or a negative control for 24 h were detected by qRT-PCR. (d) Western blot analysis of SOCS6 levels in HL-1 cells transfected with miR-19b mimic, anti-miR-19b, or a negative control for 24 h. Phosphorylated DRP1 levels in (e) whole cell lysates and in (f) mitochondrial fractions were analyzed by western blot assays. VDAC1 as a mitochondrial marker. (g) HL-1 cardiomyocytes were treated with H/R at the times indicated. Relative miR-19b levels were detected by qRT-PCR. HL-1 cardiomyocytes were transfected with miR-19b mimic or a negative control (miR-NC) and then exposed to H/R. (h) Lactate dehydrogenase (LDH) release in HL-1 cardiomyocytes. (i) Total cell death was assessed using MTT cell viability assay. (j) Apoptosis analyzed by percentage of TUNEL-positive cells (TUNEL: green; DAPI: blue). (k) Mitochondria were stained by MitoTracker Red and the percentages of fragmented mitochondria are shown. Nuclei were visualized with DAPI (MitoTracker Red: red; DAPI: blue). ^∗^*P* < 0.05 and ^∗∗^*P* < 0.01.

We next considered whether miR-19b could control the regulation of mitochondrial fission and apoptosis by repressing Socs6 expression in a similar way to Sh-Socs6. We mutated the predicted target site of miR-19b in the 3′-UTR of Socs6 mRNA and performed a luciferase reporter assay in HEK293T cells (Figures 3(a) and 3(b)). We observed that relative luciferase activity for the wild-type 3′-UTR of Socs6 was significantly lower in the presence of miR-19b, whereas in the presence of anti-miR-19b, the luciferase activity was significantly higher. Additionally, it was evident that mutated 3′-UTR of Socs6 had no response to either miR-19b or anti-miR-19b. The miR-19b expression level was significantly increased in HL-1 cardiomyocytes transfected with miR-19b mimic, while its expression was decreased after transfection with anti-miR-19b, as compared to their corresponding negative control (Figure 3(c)). Western blotting of HL-1 cardiomyocytes transfected with miR-19b mimic or anti-miR-19b confirmed that when miR-19b is overexpressed, levels of SOCS6 were lower, whereas they are higher when miR-19b was silenced (Figure 3(d)). Additionally, we also assessed the p-DRP1 and DRP1 levels in whole cell lysates (Figure 3(e)) and mitochondrial fractions (Figure 3(f)). miR-19b overexpression significantly increased p-DRP1 levels, whereas silencing of miR-19b significantly decreased pDRP1 levels in both the samples. Further, this was evident also in the assessment of pDRP1/DRP1 ratio levels. When HL-1 cardiomyocytes were subjected to H/R, the relative miR-19b levels detected by qRT-PCR were found to gradually decrease over 12 h to levels significantly lower than those under normal conditions (Figure 3(g)). The release of LDH was significantly decreased in cells transfected with miR-19b mimic and subjected to H/R compared to cells exposed to H/R (*P* < 0.05) (Figure 3(h)). As shown in Figure 3(i), H/R significantly decreased cell viability but overexpression of miR-19b could significantly rescue this defect. Moreover, in HL-1 cardiomyocytes transfected with miR-19b mimic and then exposed to H/R, apoptosis and level of fragmented mitochondria are all significantly lower compared with the negative control (*P* < 0.05) (Figures 3(j) and 3(k)), indicating that cells are subjected to less injury after H/R when miR-19b is overexpressed. Overall, these results suggest that miR-19b suppresses Socs6 and the overexpression of miR-19b generates a phenotype similar to that found with Sh-Socs6.

### 3.4. Restoration of Socs6 Reverses miR-19b-Mediated Inhibition of Mitochondrial Fission and Cardiomyocyte Apoptosis

**Figure 4:**
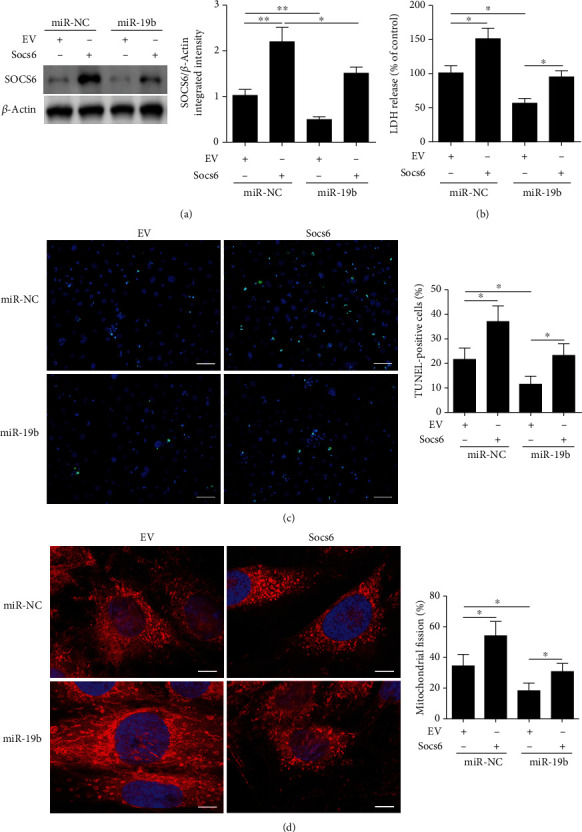
Restoration of Socs6 reverses miR-19b-mediated inhibition of mitochondrial fission and cardiomyocyte apoptosis. HL-1 cells were cotransfected with miR-19b mimic and Socs6 overexpression vectors for 24 h or miR-NC and control empty vector (EV). (a) Western blot analysis of SOCS6 levels. (b) Release of lactate dehydrogenase (LDH) from HL-1 cardiomyocytes after hypoxia and reoxygenation (H/R). (c) Apoptosis calculated by the percentage of TUNEL-positive cells (TUNEL: green; DAPI: blue). (d) Percentage of cardiomyocytes with fragmented mitochondria. Mitochondria were stained with MitoTracker Red. Nuclei were visualized by DAPI (MitoTracker Red: red; DAPI: blue). ^∗^*P* < 0.05 and ^∗∗^*P* < 0.01.

We next examined whether the restoration of Socs6 could reverse miR-19b-mediated inhibition of mitochondrial fission and apoptosis in HL-1 cells. Following H/R, the overexpression of Socs6 was found to increase the release of LDH, apoptosis, and percentage of cardiomyocytes with fragmented mitochondria (Figures 4(a)–4(d)). However, the overexpression of miR-19b can significantly alleviate the increase in damage to cells caused by overexpressing Socs6. These results indicate that the restoration of Socs6 reverses the miR-19b-mediated inhibition of mitochondrial fission and cardiomyocyte apoptosis, which is further evidence of an association between Socs6 and miR-19b.

### 3.5. miR-19b Suppresses Myocardial Infarction and Apoptosis *In Vivo*

**Figure 5:**
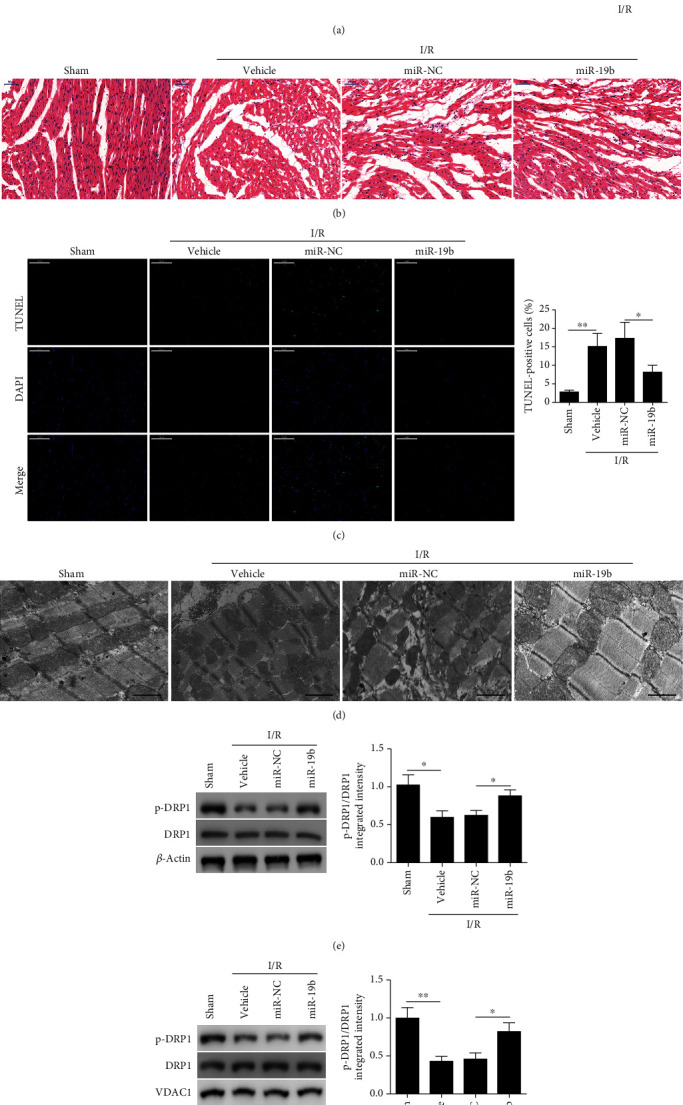
miR-19b suppresses myocardial infarction and apoptosis *in vivo*. The hearts of adult male C57BL/6 mice were injected with miR-19b. After 72 h, they were exposed to 30 min of myocardial ischemia, followed by indicated time of reperfusion. (a) Representative sections of myocardial infarction assayed by TTC staining after 24 h of reperfusion. The white arrow indicated the infarcted area. Infarct size is presented as a percentage of the area at risk. (b) Representative images of heart sections stained with hematoxylin and eosin. Scale bar, 50 *μ*m. (c) Apoptotic cells were detected by TUNEL assay after 3 h of reperfusion. Scale bar, 50 *μ*m. (d) Representative transmission electron microscopy images of mitochondrial morphology after 3 h of reperfusion. Scale bar, 1 *μ*m. Phosphorylated DRP1 levels in (e) whole heart and in (f) mitochondrial fractions were analyzed by western blot assays. VDAC1 as a mitochondrial marker. ^∗^*P* < 0.05 and ^∗∗^*P* < 0.01.

After establishing that miR-19b suppresses damage to mitochondrial fragmentation and apoptosis *in vitro*, we determined whether it could suppress myocardial infarction and apoptosis in a mouse model of I/R injury *in vivo*. miR-19b was injected into the hearts of adult male C57BL/6 mice which were then exposed to MI by ligating the coronary artery for 30 min. After 24 h perfusion, mice were euthanized and the infarct size was analyzed using planar morphometry of heart sections stained with TTC. Representative sections of myocardial infarction assayed by TTC staining are shown in Figure 5(a). Infarct size was significantly reduced in mice that had received miR-19b. Moreover, there were less apoptotic cells in the myocardium of mice receiving miR-19b and TEM images revealed that mitochondria were less fragmented (Figures 5(b)–5(d)). Additionally, we performed western blotting analysis of DRP1 in the whole heart (Figure 5(e)) and in mitochondrial fractions (Figure 5(f)). Under I/R injury, mice that received miR-19b had significantly higher pDRP1 levels compared to its controls. Further, these results were also evident from the pDRP1/DRP1 ratio. These results support *in vitro* findings; miR-19b also suppresses myocardial infarction and apoptosis *in vivo*.

### 3.6. QK Participates in the Regulation of miR-19b Expression

**Figure 6:**
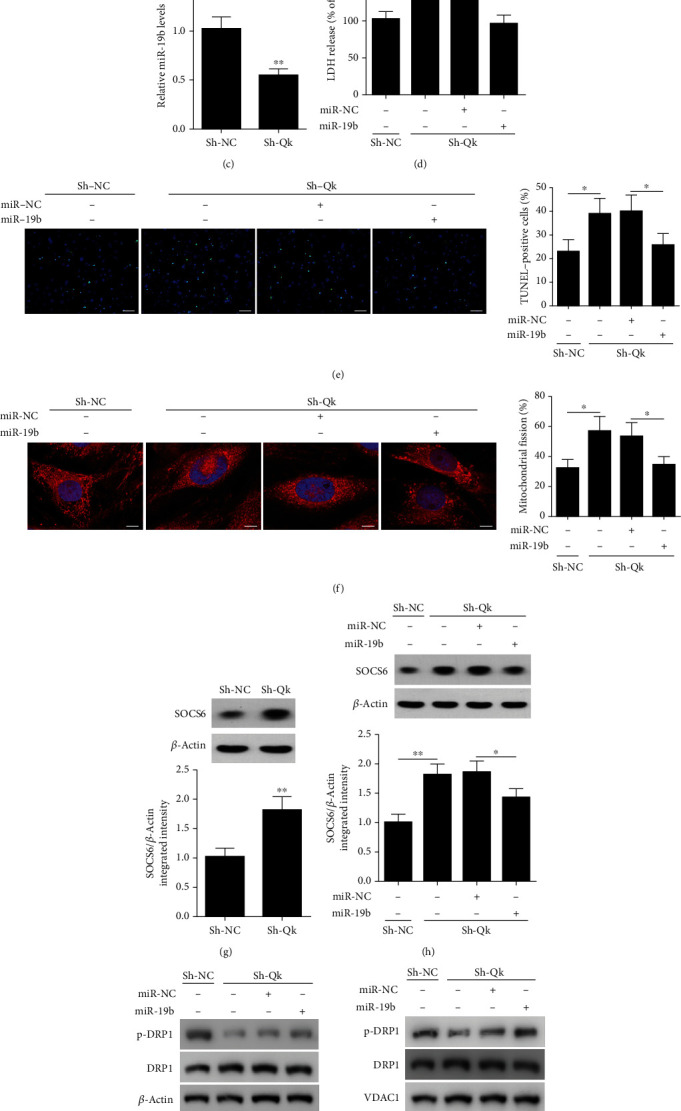
QK participates in the regulation of miR-19b expression. (a) HL-1 cardiomyocytes were infected with lentiviral constructs of QK ShRNA or a negative control (Sh-NC). Relative QK mRNA levels were detected by qRT-PCR. (b) QK levels were analyzed by western blotting. (c) Relative miR-19b levels were detected by qRT-PCR. (d) Release of lactate dehydrogenase (LDH) from HL-1 cardiomyocytes exposed to hypoxia and reoxygenation (H/R). (e) Representative images of apoptosis analyzed by TUNEL staining (TUNEL: green; DAPI: blue). Percentages of TUNEL-positive cells are shown. (f) Representative confocal images of mitochondrial morphology in cells stained with MitoTracker Red. Percentage of fragmented mitochondria in cardiomyocytes is shown. Nuclei were visualized by DAPI (MitoTracker Red: red; DAPI: blue). (g) Western blotting analysis of SOCS6 levels in HL-1 cells infected with lentiviral constructs of QK ShRNA or its negative control (Sh-NC). (h) Lentiviral constructs of QK ShRNA-infected HL-1 cells were transfected with miR-19b mimic for 24 h, and then, SOCS6 levels were detected by western blotting. Phosphorylated DRP1 levels in (i) whole cell lysates and in (j) mitochondrial fractions were analyzed by western blot assays. VDAC1 as a mitochondrial marker. ^∗^*P* < 0.05 and ^∗∗^*P* < 0.01.

Finally, we assessed whether QK may participate in the regulation of miR-19b. HL-1 cardiomyocytes were infected with lentiviral constructs of QK ShRNA or a negative control, and QK mRNA and protein levels were confirmed to be suppressed by qRT-PCR and western blotting, respectively (Figures 6(a) and 6(b)). Relative miR-19b levels were then measured by qRT-PCR (Figure 6(c)), which indicated that suppressing levels of QK reduced the expression of miR-19b. Moreover, following H/R, silencing QK significantly increases LDH release, the number of apoptotic cells, and mitochondrial fission but overexpressing miR-19b reverses this effect (Figures 6(d)–6(f)). We also assessed the influence of QK on SOCS6 levels in HL-1 cells infected with lentiviral constructs of QK ShRNA (Figure 6(g)). Higher levels of Socs6 were observed when QK was silenced, but the overexpression of miR-19b reversed this trend (Figure 6(h)). We also performed western blotting analysis of DRP1 in the whole cell lysates (Figure 6(i)) and in mitochondrial fractions (Figure 6(j)). Cells silenced for QK displayed a significantly lower level of p-DRP1; however, overexpression with miR-19b clearly rescued and increased p-DRP1 levels. Further, these results were also evident from the pDRP1/DRP1 ratio. Overall, these results suggest a QK/miR-19b/Socs6 pathway, whereby miR-19b can suppress Socs6 but is in turn regulated by QK.

## 4. Discussion

The disruption of mitochondrial dynamics during myocardial I/R injury presents problems in clinical practice and may contribute to the severity of the infarct size [28, 29]. Inhibiting mitochondrial fission is thought to protect the heart against I/R injury [13, 15], and the delivery of mitochondria to cardiovascular tissue by vascular perfusion has been successfully applied in animal models to significantly reduce the infarct size [30, 31]. SOCS6 forms a complex with DRP1, a GTPase of the dynamin superfamily that is essential for mitochondrial fission, and phosphoglycerate mutase 5 (PGAM5), a mitochondrial phosphatase, to facilitate DRP1 mitochondrial translocation [18]. The upregulation of Socs6 is known to have a detrimental effect on mitochondrial function by inhibiting the phosphorylation of DRP1 [18].

In the present study, we assessed the impact of Socs6, QK, and miR-19b expression on mitochondrial morphology in I/R injury using cardiomyocytes and a murine model. Consequently, we found that Socs6 downregulation led to increased DRP1 phosphorylation and a fewer percentage of fragmented mitochondria and that the silencing of Socs6 was able to reduce the increases in the levels of mitochondrial fragmentation. Downregulated Socs6 expression also led to a decreased number of apoptotic cells and lowered cell injury. Moreover, in a mouse model of I/R injury, the infarct size was greater in mouse hearts expressing Sh-NC than in those with Socs6 silenced. Nishimura et al. [32] reported that DRP1 interacts with actin-binding protein filamin A through guanine nucleotide exchange to mediate myocardial senescence via mitochondrial fission in mice after MI. They found that hypoxic stress promoted the interaction of filamin A with the GTPase domain of DRP1 which consequently led to increased actin binding by DRP1, and they suggest that developing an inhibitor of DRP1 may be useful in the management of I/R injury. Lin et al. [33] first studied SOCS6 because its inactivation occurred frequently in gastric cancer. In later studies, it was found that it promotes intrinsic apoptosis by targeting mitochondria and inducing fragmentation through its interaction with DRP1 [18]. Our results support these findings; H/R was severely detrimental to the number of viable cardiomyocytes. However, downregulating Socs6 in cardiomyocytes resulted in significantly fewer apoptotic cells and a reduced the percentage of fragmented mitochondria.

In this study, we found that miR-19b targeted and downregulated Socs6 in cardiomyocytes, which led to less injury and fragmented mitochondria. We chose to study miR-19b because it was the only member of the miR-17-92 cluster to be downregulated in the infarct area following I/R injury [27]. miR-19 has also been found to promote the progression of osteosarcoma by targeting and downregulating Socs6 in association with Janus kinase 2 (JAK2)/signal transducer and activator of transcription 3 (STAT3) signaling [34]. The JAK2/STAT3 signaling pathway is involved in the progression of several cancers and is associated with other SOCS proteins through feedback regulation in various cell processes [35–38]. Although the research on JAK2/STAT3 signaling and mitochondrial fragmentation is limited, one study has proposed that JAK2/STAT3 signaling can prevent myocardial I/R injury by reducing I/R-induced mitochondrial oxidative damage [39]. Given this association with I/R injury and mitochondrial oxidative damage, the regulation of Socs6 by miR-19 in association with JAK2/STAT3 signaling would be worthy of further investigation. In microglia, miR-19 is also known to inhibit oxidative stress and cell apoptosis by its association with PTEN/PI3K/Akt pathway-related proteins [40]. The numerous target genes associated with miR-19 may be due to noncanonical binding related to the RBP Hu antigen R (HuR). In a study screening for miRNA involved in breast cancer therapy resistance, UTR sequences responded to miR-19b despite lacking a canonical binding site; however, the sequences contained a consensus site for HuR [41]. It may be worthwhile investigating the number of target genes associated with miR-19 during I/R to determine if any associate through noncanonical binding.

In our study, suppressing the levels of QK reduced the expression of miR-19b, which then significantly increases LDH release, the number of apoptotic cells, and mitochondrial fission. Downregulating QK also increased the expression of SOCS6. Overall, we found that miR-19b can suppress Socs6 but is in turn regulated by QK.

QK deficiency was found to increase susceptibility to I/R injury in a mouse model of diabetes [42]. The Quaking gene is known to exist in three alternative isoforms, QKI-5, QKI-6, and QKI-7, which can dimerize with each other [22]. The isoforms all share the same RNA-binding domain but have different amino acid sequences at the C-terminus and can translocate between the nucleus and cytoplasm. In the diabetic model mentioned previously, the deficiency of Qki5 overactivated forkhead box O1 (FoxO1), which increases sensitivity to stress and contributed to I/R injury whereas Qki5 overexpression destabilized FoxO1 mRNA. In another study, miR-208a/b was found to exacerbate H/R injury by downregulating Qki5 and Qki6 in cardiomyocytes [43]. Therefore, QKI downregulation seems to be a recurring factor in susceptibility to I/R injury. The ratio and involvement of different isoforms may be important and are worthy of further investigation as different isoforms could be involved in the regulation of miR-19b.

## 5. Conclusion

In agreement with other studies [13, 15], the present study found that the inhibition of mitochondrial fission attenuated the damage sustained after I/R. This inhibition of mitochondrial fission through downregulating Socs6 via a QK/miR-19b/Socs6 axis is an intricate process that may even involve noncanonical interactions and could form the basis of future research.

## Data Availability

The data used to support the findings of this study are available from the corresponding authors upon request.

## Abbreviations

H/R: Hypoxia and reoxygenation
QK: Quaking
MI: Myocardial infarction
I/R: Ischemia/reperfusion
DRP1: Dynamin-related protein 1
SOCS6: Suppressor of cytokine signaling 6
STAR: Signal transduction and activation of RNA
RBPs: RNA-binding proteins
miRNA: MicroRNA
UTR: Untranslated regions
LDH: Lactate dehydrogenase
TUNEL: Terminal deoxynucleotidyl transferase-mediated dUTP nick end labeling
TTC: Triphenyltetrazolium chloride
PGAM5: Phosphoglycerate mutase 5
JAK2: Janus kinase 2
STAT3: Signal transducer and activator of transcription 3
HuR: Hu antigen R
FOXO1: Forkhead box O1.
